# Heat exposure improves acute copper tolerance in the intertidal copepod *Tigriopus californicus*

**DOI:** 10.1007/s11356-026-37621-2

**Published:** 2026-03-25

**Authors:** Alice L. Coleman, Suzanne Edmands

**Affiliations:** Department of Biological Sciences, University of Southern California, 3616 Trousdale Parkway #130, Los Angeles, CA 90089 USA

**Keywords:** Cross-protection, Multiple stressors, Stress tolerance, Copper, Heat, Oxidative stress

## Abstract

Cross-protection, the phenomenon of exposure to one stressor conferring temporarily increased resistance to a second stressor, is a key mechanism that may help organisms cope with multiple stressor challenges. Here, we tested for cross-protection between acute copper and heat stresses in the intertidal copepod *Tigriopus californicus*, which exhibits local adaptation to the Northern Hemisphere’s latitudinal thermal gradient. Curiously, copper tolerance in this species also increases at lower latitudes, although there is little evidence for a latitudinal gradient of copper pollution. Because both heat and copper induce oxidative stress, we hypothesized that the co-occurring tolerance patterns are driven by a shared molecular response mechanism that could also generate cross-protection during sequential stressor exposure. To test this hypothesis, we measured the acute tolerances of seven allopatric *T. californicus* populations after isolated and sequential stress exposures and analyzed the transcriptomic responses of the most geographically distant populations to both stressors. We observed unidirectional cross-protection between copper and heat, wherein acute copper tolerance increased when copepods were exposed to heat prior to toxicity testing, but not when stressor order was reversed. Gene expression during exposure to both stressors was highly population-specific, but there were commonalities in how the populations modulated the oxidative defense system, catabolic reactions, and other processes in response to each stressor that could contribute to the observed cross-protection. Together, these findings highlight how shared stress‐response pathways can shape tolerance to sequential environmental challenges, with implications for predicting organismal resilience in a changing climate.

**Supplementary Information:**

The online version contains supplementary material available at 10.1007/s11356-026-37621-2.

## Introduction

Multiple stressors are becoming the norm in aquatic ecosystems. Natural and anthropogenic forces such as climate change, habitat degradation, overexploitation, and chemical pollution frequently co-occur spatially and temporally, affecting much of the global ocean (Halpern et al. 2015) and jeopardizing aquatic biodiversity. These various stressors can interact in complex and unexpected ways, making understanding the combined effects of multiple stressors a major challenge (Côté et al. 2016; Piggott et al. 2015). The scale of this task is increased by the intensification of disturbances to the environment that are forecasted to occur with climate change (Tilman et al. 2017), meaning there is an urgent need for research to evaluate and predict how aquatic ecosystems will cope with the cumulative effects of multiple stressors.

An environmental factor becomes a stressor when it disturbs an organism’s homeostasis to such an extent that fitness is compromised (Schulte 2014). Stressors can trigger diverse physiological and molecular mechanisms, leading to several possible outcomes when an organism encounters more than one stressor. Classic theory predicts that exposure to multiple stressors will result in either an additive, synergistic, or antagonistic response (Folt et al. 1999). Stressors are considered additive when the cumulative effect of a group of stressors equals the sum of the effects of each individual component. Synergistic stressors amplify each other such that the combined effect exceeds the predicted additive effect, while antagonistic stressors dampen each other, resulting in a smaller than expected combined response. Given that synergistic stressors may result in amplified stress responses, the identification of synergistic combinations has been highly emphasized in stress biology literature (Côté et al. 2016). However, frameworks for understanding when each type of interaction may occur are valuable when designing and implementing conservation actions. Knowledge of antagonistic stressor combinations is particularly important in this context as management plans might be ineffective or even detrimental if antagonistic stressors are assumed to be synergistic (Brown et al. 2013). Antagonistic outcomes can arise in multiple ways, such as when one stressor removes the most sensitive individuals from a population, when one stressor mitigates the effects of others, or when exposure to one stressor induces protective mechanisms that influence responses to subsequent challenges (Breitburg & Riedel 2005; Rodgers & Gomez Isaza 2021). Whether a combination of stressors results in an additive, synergistic, or antagonistic effect depends in part on the timing and intensity of the stressors (Brooks & Crowe 2019; Gunderson et al. 2016). Because the magnitude of a stress event determines whether it exceeds an organism’s physiological threshold, its intensity can dictate whether a protective response is activated or damage occurs. Likewise, the relative timing of stress events becomes critical when stressors are temporally offset, as exposure to an initial stressor can modify an organism’s response to subsequent challenges (Gunderson et al. 2016). This sequence-dependent effect can produce either transiently increased tolerance (“cross-protection”) or heightened vulnerability (“cross-susceptibility”) to a subsequent stressor (Todgham & Stillman 2013).

Cross-protection has been documented across the diversity of life for a wide range of stressor combinations (Rodgers & Gomez Isaza 2023). For example, Todgham et al. (2005) demonstrated that the survival of tidepool sculpins in severe osmotic and hypoxic conditions increased when the fish experienced a heat shock prior to exposure. Similarly, Hůla et al. (2022) found that acclimation to drought conditions improved the freeze tolerance of larvae of the drosophilid fly *Chymomyza costata*. Cross-protection is hypothesized to arise from a priming effect, wherein an initial stressor activates either the same protective mechanisms (referred to as “cross-tolerance”) or signaling pathways that trigger independent protective mechanisms (referred to as “cross-talk”) as subsequent stressors (Rodgers & Gomez Isaza 2023). In contrast, during cross-susceptibility, the initial stressor either overwhelms or impairs protective mechanisms, leaving an organism less capable of coping with later challenges (Todgham & Stillman 2013). Because individuals that exhibit cross-protection may possess fitness advantages during extreme climactic events or exposure to novel combinations of stressors, it has been hypothesized to serve as a possible “pre-adaptation” that could buffer species from future threats (Ramegowda et al. 2020; Rodgers & Gomez Isaza 2023). In this study, we explore the possibility of cross-protection arising in the model marine copepod *Tigriopus californicus* during sequential exposures to copper (Cu) and high heat.

*T. californicus* is a small crustacean that inhabits splash pools in the high intertidal zone along the Pacific coast of North America from Baja California to Alaska (Edmands 2001; Ganz & Burton 1995). Substantial genetic divergence occurs between *T*. *californicus* populations at multiple spatial scales (R. Burton et al. 1979; Edmands 2001; Willett & Ladner 2009), and populations exhibit local adaptation to multiple abiotic environmental variables. The most prominent example of this local adaptation occurs for temperature, for which several studies have found that populations from lower latitudes exhibit higher thermal tolerance than their northern counterparts (Kelly et al. 2011; Leong et al. 2018; Willett 2010). Similar latitudinal clines in tolerance have also been identified for salinity, which frequently covaries with temperature, and more surprisingly, for copper (Lee et al. 2021; Leong et al. 2018; Sun et al. 2015). Specifically, Sun et al. (2015) found that the maximum temperature at a *Tigriopus* population’s collection site was positively correlated with its acute copper tolerance, meaning that populations from warmer, southern areas are more tolerant of copper than populations from cooler northern areas.

Copper pollution is prevalent in coastal regions (Balls 1985; Han et al. 1994; Ozseker et al. 2013), but unlike temperature, is generally not known to increase predictably with decreasing latitude. To the best of our knowledge, there are currently no published measurements of dissolved copper concentrations in *T. californicus* habitat that demonstrate a comparable latitudinal gradient in copper pollution comparable to the species’ well established thermal cline. Preliminary environmental sampling referenced by Sun et al. (2015) (P. Sun, unpublished data) suggested that dissolved copper levels in *T. californicus* splash pools were comparable to coastal concentrations, whereas their experimentally derived copper tolerance values exceeded surface water concentrations by several orders of magnitude. Because of these discrepancies, Sun et al. (2015) went on to hypothesize that the latitudinal trend in copper tolerance may represent an exaptation derived from a shared defense mechanism with heat stress rather than a direct adaption to copper exposure. The possibility of a shared molecular and physiological response to these heterologous stressors arises from the induction of oxidative stress by both copper and heat.

Copper is an ubiquitous pollutant in the marine environment, arising from anthropogenic sources such as antifouling paints, mining, and industrial discharge (Brooks & Waldock 2009). In living systems, copper plays paradoxical dual roles as both an essential trace metal and potent toxicant. During normal conditions, free copper ions are virtually nonexistent in cells, as most copper atoms are sequestered in different enzymes that perform important roles in routine physiology (Harrison et al. 1999; Tapia et al. 2004). Toxicity occurs when the intracellular copper concentration overwhelms the storage system (Harrison et al. 1999; Tapia et al. 2004), enabling excess copper ions to accumulate and participate in a Fenton-like reaction with hydrogen peroxide to produce reactive oxygen species (ROS; Pham et al. 2013). Like copper, ROS perform important physiological functions at low concentrations but become hazardous at high concentrations by oxidatively damaging integral cellular structures and processes (Dröge 2002; Kong & Lin 2010; Lesser 2006). The ROS concentration in a cell is dynamically balanced between their production and elimination, and when this equilibrium is disrupted in favor of production, a cell is said to be experiencing oxidative stress. Many environmental stressors, including both copper and heat, are known to induce oxidative stress in aquatic organisms through a variety of mechanisms (Lushchak 2011). Heat, a critical stressor in marine environments, initiates oxidative stress as a byproduct of by increasing the rates of all chemical reactions in a cell, including respiration. Reactive oxygen species such as superoxide $O_2^{-\bullet}$ and hydrogen peroxide (H_2_O_2_) are natural byproducts of oxidative phosphorylation (Belhadj Slimen et al. 2014), meaning that heat-driven increases in respiration also increase ROS production.

Aerobic organisms possess a robust defense system to combat the deleterious effects of ROS. This system is composed of both enzymatic and non-enzymatic molecules that act to neutralize ROS and prevent or repair damage to proteins, lipids, and nucleic acids. Major antioxidant enzymes include superoxide dismutase (SOD), which catalyzes the breakdown of superoxide into molecular oxygen (O_2_) and hydrogen peroxide, as well as catalase (CAT) and glutathione peroxidase (GPx) which both convert hydrogen peroxide into water (Sies 1993). Non-enzymatic antioxidants such as the tripeptide glutathione (GSH), carotenoids, and α-tocopherol (Vitamin E) contribute to oxidative defenses by neutralizing ROS via reduction. Heat shock proteins (HSPs) are also known to respond to oxidative stress as molecular chaperons, assisting in the correct folding of polypeptide chains and preventing the aggregation of misfolded proteins affected by ROS (Ghosh et al. 2018). Both heat and copper significantly modify the expression and activity of HSPs and antioxidants in *Tigriopus* (Kim et al. 2014; A. J. Li et al. 2023; Rhee et al. 2009; Schoville et al. 2012), suggesting the response mechanisms for these heterologous stressors may have shared features. As a result, components of the thermal stress response mechanism could potentially be co-opted to alleviate oxidative damage caused by copper, and copper-induced oxidative defenses could similarly contribute to thermal tolerance.

Here, we expand upon studies of the latitudinal patterns of local adaptation in *T. californicus* and investigate whether this species exhibits cross-protection between copper and heat. Using seven allopatric populations from California, USA, we conducted a series of acute stress assays to measure each population’s tolerance of both stressors, first when experienced individually and then after a prior exposure from the other stressor. The in vivo experiments were paired with exploratory transcriptomic sequencing of the two most geographically distant populations to evaluate gene expression responses to each stressor. These combined approaches enabled us to explicitly test for cross-protection between copper and heat and to investigate the underlying response mechanisms for each stressor, shedding light on multiple stressor tolerance in *T. californicus*.

## Methods

**Fig. 1:**
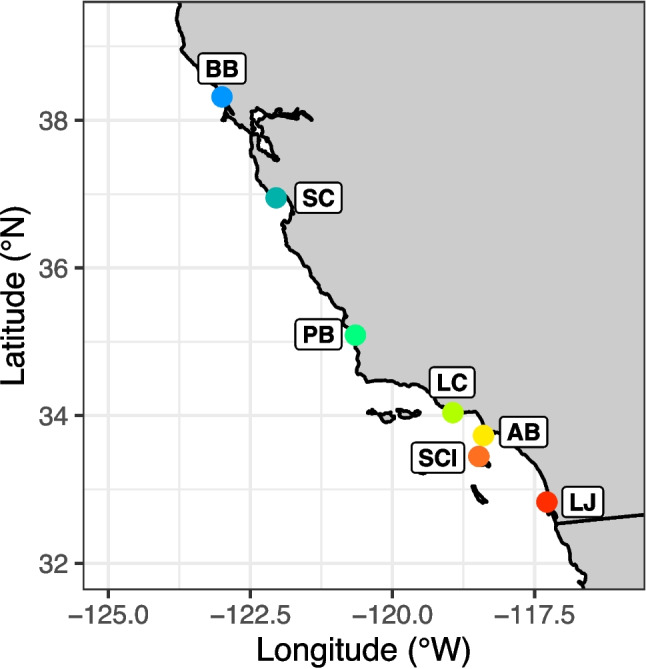
Locations of the seven T. californicus populations sampled in California. BB, Bodega Bay; SC, Santa Cruz; PB, Pismo Beach; LC, Leo Carrillo; AB, Abalone Cove; SCI, Santa Catalina Island; LJ, La Jolla

*T. californicus* were sampled from supratidal splash pools at seven sites in California, USA (Fig. 1; Table S1), with each population collected on a single occasion. Five populations were collected in November 2022: Bodega Bay (BB), Santa Cruz (SC), Abalone Cove (AB), Santa Catalina Island (SCI), and La Jolla (LJ). We performed an additional round of sampling in February 2024 at Pismo Beach (PB) and Leo Carrillo State Beach (LC) to improve latitudinal resolution of the study. Sampling sites were concentrated in California because there is greater genetic and phenotypic variation among *T. californicus* populations located south of Oregon than those in more northern areas (Edmands 2001). Following laboratory acclimation, the five populations collected in November 2022 were assayed between January 2023 and September 2024, while the two populations collected in February 2024 were assayed between March and September 2024.

Copepods were collected directly from splash pools using a large volume pipette and transferred into 1-L Nalgene bottles. Two bottles were collected at each site and transported from the field to the University of Southern California (USC), where the contents of each bottle were poured through a 150-μm mesh sieve. Copepods were briefly rinsed with 37-μm triple-filtered natural seawater sourced from the Wrigley Marine Science Center on Santa Catalina Island and transferred into 1-L glass beakers (Carolina Biological) containing 800 mL of the filtered water. Cultures were initially provided with a mixture of TetraMin fish food (Tetra Holding Inc., USA) and the blue-green algae Spirulina (Nutraceutical Science Institute, USA) at a concentration of 0.1 g of each food per liter seawater. After this initial feeding, copepods were allowed to feed freely on naturally growing algae in their beakers, with additional TetraMin and Spirulina added only if water levels decreased, or algal growth appeared insufficient. Cultures were incubated at 20 °C with a 12-h light:dark cycle and allowed to acclimate to laboratory conditions for a minimum of 4 weeks before stress testing. All cultures remained healthy and active throughout acclimation, and each population produced sufficient individuals for all experiments.

### Acute copper toxicity assays

**Fig. 2:**
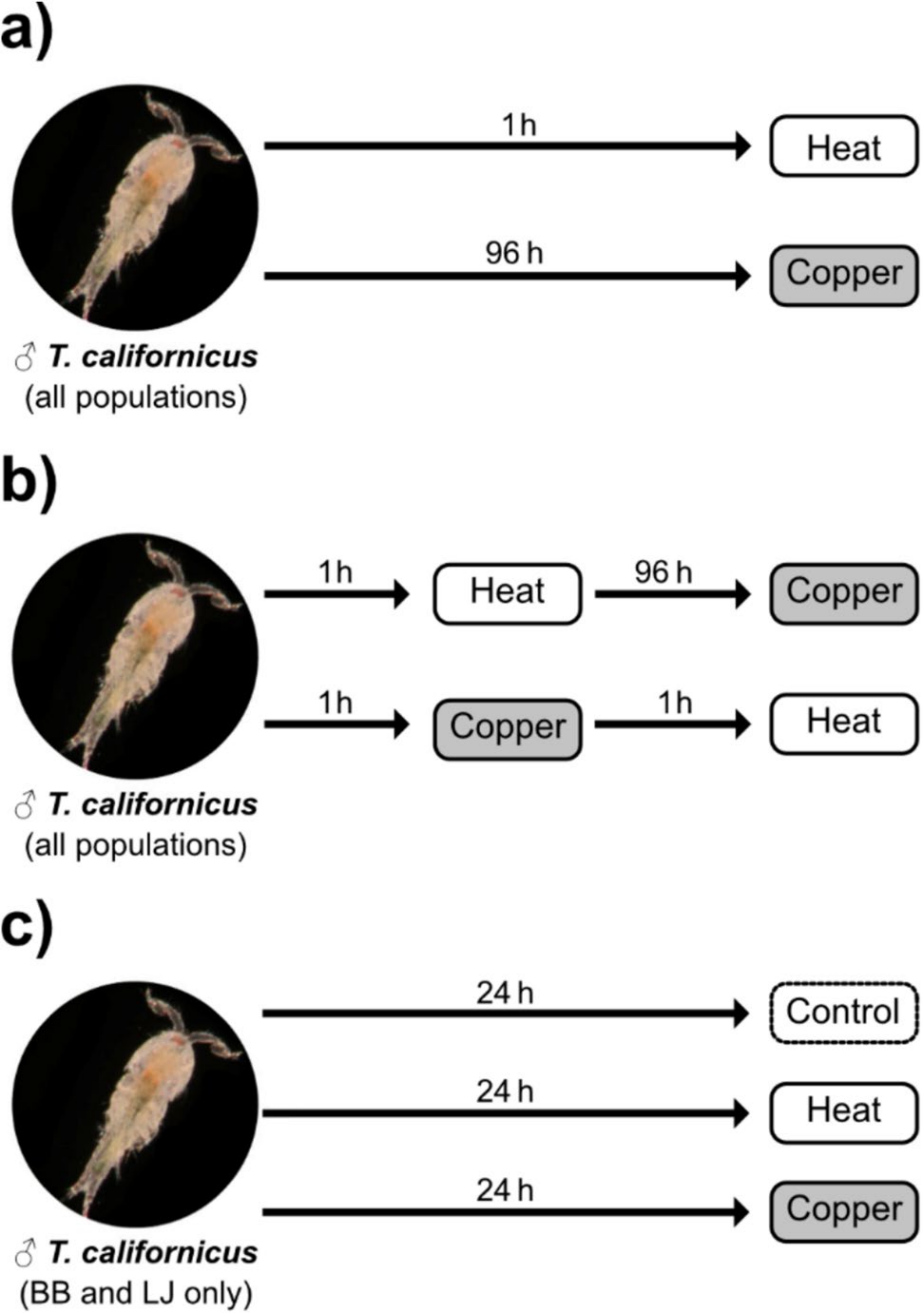
Basic design of single stressor exposures (**a**), sequential stressor exposures (**b**), and exposures for animals used for RNA-seq (**c**). Male *T*. *californicus* image in all panels adapted with permission from Tsuboko-Ishii & Burton (2018)

Acute copper tolerance was measured as a 96-h median lethal concentration (LC50), which represents the concentration of a chemical required to kill half of a test population over a 96-h period (Fig. 2a). In these assays, 12 adult males from the same population were individually distributed at random into wells of polystyrene 24-well plates (BD Falcon) containing 2 mL of Cu test solution. Multiple populations were tested concurrently on separate plates. Animals were checked every 24 h for mortality until a total of 96 h lapsed, with mortality assigned when individuals did not respond to gentle prodding from a probe. If the Cu test solution did not produce complete mortality (12 deaths) of a plate, then that concentration was re-tested at least twice to improve LC50 estimation. Male *T. californicus* were used because they are generally less stress tolerant than females (Willett 2010). No feeding or solution renewal took place during these assays.

Cu test solutions were prepared with solid CuSO_4_•5H_2_0 (Sigma) dissolved in 37-μm triple-filtered autoclaved seawater (FASW) sourced from the Wrigley Marine Science Center on Santa Catalina Island in 12 concentrations: 0, 5, 10, 20, 40, 60, 80, 100, 120, 180, 250, and 500 mg/L Cu. Stock solutions were prepared on an as-needed basis and stored in capped 500-mL glass media bottles (Corning Pyrex) at room temperature. Stock solution bottles were inverted multiple times to ensure resuspension of copper immediately prior to use. All glassware and plastic items utilized in toxicity tests were acid-washed overnight in 0.1 M HCl and rinsed with deionized water between uses.

### Acute heat stress tests

Acute heat tolerance was measured as median lethal temperature (LT50), which represents the temperature that kills half of a test population (Fig. 2a). For each assay, 12 adult males from the same population were individually distributed into the wells of one row of a polyvinyl chloride 96-well plate (BD Falcon) containing 50 μL of triple-filtered FASW. Multiple populations were assayed concurrently, with individuals from the same population assigned to the same row of a plate and their positions randomized within rows. Heat stress was initiated by a PTC-200 thermal cycler (MJ Research) which was programmed to ramp from 20 °C to the target temperature at a rate of 0.1 °C per minute. Plates were then held at the target temperature for 1 h, followed by a 1-h recovery period at 20 °C. Assays were repeated in increments of 1 °C with fresh animals until complete mortality in a plate was observed. Stress temperatures that elicited incomplete mortality (< 12) in a plate were repeated at least twice. Experimental stress temperatures ranged between 35 and 42 °C, which are consistent with maximum temperatures observed in *T. californicus* splash pools during natural heating events (Leong et al. 2018).

### Heat-to-copper sequential assays

To assess the effect of heat exposure on acute copper tolerance, we conducted a modified LC50 experiment in which copepods experienced a 1-h long “heat shock” (35 °C) prior to undergoing a copper toxicity test (Fig. 2b). In these experiments, 12 adult males from the same population were individually distributed at random into the wells of 24-well plates that contained 2 mL of triple-filtered FASW, incubated at 35 °C for 1 h and allowed to recover for 1 h at 20 °C. Multiple populations were tested concurrently on separate plates. Following recovery, individuals were confirmed to be alive by their response to gentle prodding with a probe before being transferred into new 24-well plates for a 96-h LC50 assay that was conducted as described above. We selected 35 °C for the heat shock treatment because this temperature represents the lower bound of stress temperatures used in the LT50 assays and has been applied as a sublethal heat shock in other *Tigriopus* studies (Harada & Burton 2020; Schoville et al. 2012).

### Copper to-heat sequential assays

To assess the effect of copper exposure on acute heat tolerance, we conducted a modified LT50 experiment in which copepods experienced a 1-h long “copper shock” (60 mg/L Cu) before undergoing a heat stress assay (Fig. 2b). For each assay, 12 adult males from a given population were individually distributed into the wells of one row of a 96-well plate containing 50 μL of 60 mg/L Cu solution per well. Multiple populations were assayed concurrently on a single 96-well plate, with individuals from the same population assigned to the same row and their positions randomized within rows.

Plates were held at 20 °C incubator for 1 h, and then animals transferred into a new 96-well plate containing 50 μL of clean FASW per well and allowed to recover for 1 h at 20 °C. Following recovery, animals were confirmed to be alive by their response to gentle prodding with a probe before being placed into the thermocycler for an LT50 test conducted as described above. We used 60 mg/L Cu in these assays as it was expected to be sufficiently strong to elicit a physiological response in the short exposure period.

### Stress data analyses

Population LC50s and LT50s were calculated in R with a generalized linear model using a binomial distribution and the dose.p() function from the package MASS (Venables and Ripley 2002; Team 2024). Post hoc comparisons of each population’s tolerance values from the sequential and single stressor assays were conducted using the ratio_test() function obtained from the package ecotox (Wheeler et al. 2006), with significance determined when *p* < 0.05. Linear regression analyses were performed to evaluate the relationships of the LC50 and LT50 data with latitude, as well as the relationship between the LC50s and LT50s. Quadratic regression models were also evaluated to assess potential nonlinear relationships within the data. Linear and quadratic model fits were compared using the Akaike Information Criterion (AIC).

#### RNA isolation, library preparation, and sequencing

RNA-sequencing (RNA-seq) was performed for separate pooled samples of adult male copepods from Bodega Bay (BB) and La Jolla (LJ) following 24-h exposure to either 60 mg/L Cu or 35 °C (Fig. 2c), along unexposed controls (20 °C, no Cu). Pool size was 24 animals for the control and heat treatments and 36 animals for the copper treatment. Pooling individuals for RNA-seq has been shown to be an effective approach for differential gene expression analysis (Ko & Van Raamsdonk 2023) and has been employed previously in the *Tigriopus* transcriptomic literature (Barreto et al. 2011; Healy & Burton 2023; Schoville et al. 2012). We prepared two biological replicates of each experimental treatment and control per population, resulting in a total of 12 RNA-seq samples.

Immediately after the exposure period ended, animals were transferred without intermediate freezing into ZR BashingBead Lysis Tubes (Zymo Research) containing TRI reagent (Zymo Research) and homogenized using a TissueLyser II (Qiagen). RNA was then extracted with a Direct-zol RNA Miniprep kit (Zymo Research) according to the manufacturer’s instructions. The RNA concentration in each sample was quantified using an Invitrogen Quibit 4 Fluorometer (ThermoFisher Scientifc) and a Quibit RNA High Sensitivity Assay Kit (ThermoFisher Scientific). RNA quantity and quality metrics for each sample are provided in Table S2. Library preparation and sequencing were outsourced to Admera Health (New Jersey, USA), who constructed libraries using a NEB Next Ultra II Directional Library Prep Kit (New England Biolabs) and sequenced them in 150 bp paired end reads on an Illumina platform.

#### Read mapping and differential expression analysis

Raw read quality was evaluated with FastQC (Andrews 2010). Reads were then trimmed using Trimmomatic (Bolger et al. 2014) and secondarily evaluated with FastQC. Because the trimming did not appear to improve read quality, we proceeded to map our raw reads to the *T. californicus* reference genome for the San Diego population (Barreto et al. 2018) using HISAT2 (Putri et al. 2022). Read counts after alignment were obtained using HT-Seq (Putri et al. 2022) and then imported into R for further analyses using DESeq2 (Love et al. 2014). Principal component analysis (PCA) of the 500 genes with the highest variance in each sample after regularized log transformation was performed using plotPCA() from DESeq2 to visualize the relationships among all 12 samples together and as well as when they were grouped by population. Variance partitioning analysis was conducted using the package variancePartition (Hoffman & Schadt 2016) to quantify the contributions of population, treatment, and population × treatment interactions to gene expression.

Differential expression analysis of RNA-seq data was conducted with DESeq2 using the following design: ~ population + treatment + population:treatment. Given the low number of biological replicates (*n* = 2) per population × treatment combination, statistical power to detect subtle changes in expression is limited. However, DESeq2’s negative binomial framework estimates dispersion by sharing information across genes, enabling conservative differential expression inference under low-replicate designs (Anders & Huber 2010). Significance testing was performed using a Wald test, and multiple test corrections were performed following Benjamini and Hochberg (1995). Genes with adjusted *p* values < 0.1 were treated as significantly differentially expressed. Specific contrasts were used to evaluate the effects of population on treatment response as well as to compare the responses of the populations to each treatment, which was created by using a grouping variable for population and treatment (BB-control, BB-heat, etc.). Names of differentially expressed genes (DEGs) were extracted from the *T. californicus* annotation package retrieved from AnnotationHub (Morgan & Sheperd 2024). Following Li et al. (2020), we searched our DEG lists for antioxidant genes, heat shock proteins, and genes with “stress” or “stress-induced” in their name to determine which components of the oxidative defense system responded to copper and heat exposure. We additionally searched for genes related to chitin metabolism and cuticle formation which are known to respond to heat stress in *T. californicus* (Harada & Burton 2020; Schoville et al. 2012).

#### Gene ontology enrichment analysis

Rank-based enrichment analysis of biological process (BP) gene ontology (GO) terms was performed using the GO_MWU package (Wright et al. 2015). In this analysis, GO terms with fewer than ten associated genes are filtered out, and the remaining terms are evaluated for significant enrichment by either up- or downregulated genes using the Mann–Whitney *U* test. Redundant and highly similar GO terms are then merged according to complete linkage clustering based on the fraction of shared genes, generating a hierarchical clustering of GO categories based on the number of genes shared between them.

## Results

### Acute tolerance

**Fig. 3:**
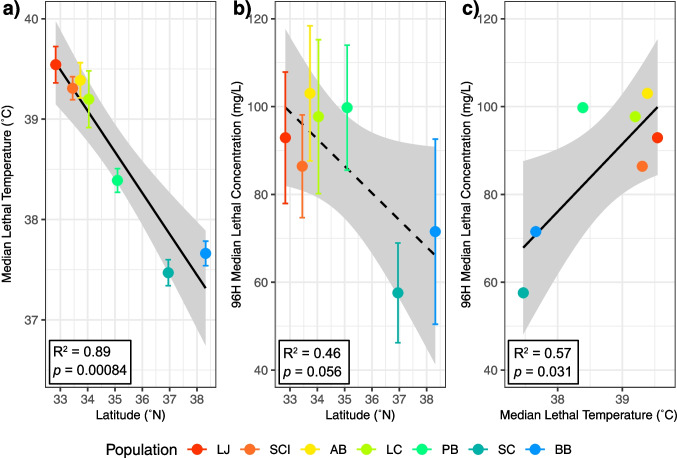
Linear regressions between latitude and population median lethal temperatures (**a**), between latitude and 96-h median lethal concentrations (**b**), and between median lethal temperature and concentration (**c**). Error bars represent the standard error of each LT50 or LC50 value. The shading around each trend line represents its 95% confidence interval. Solid trend lines are statistically significant (*p* < 0.05), dashed trend lines are not (*p* > 0.05). BB, Bodega Bay; SC, Santa Cruz; PB, Pismo Beach; LC, Leo Carrillo; AB, Abalone Cove; SCI, Santa Catalina Island; LJ, La Jolla

Acute heat tolerance decreased significantly with increasing latitude (Fig. 3a; *R*^2^ = 0.89*, p* = 0.00084). Acute copper tolerance also decreased with increasing latitude, although this trend did not reach statistical significance (Fig. 3b; *R*^2^ = 0.46*, p* = 0.056). Linear regression analysis also highlighted a significant positive association between LT50 and LC50 values (Fig. 3c; *R*^2^ = 0.57*, p* = 0.031) across populations. Quadratic models provided marginally improved fit over linear for the LT50-latitude and LT50-LC50 relationships (Table S3).

**Fig. 4:**
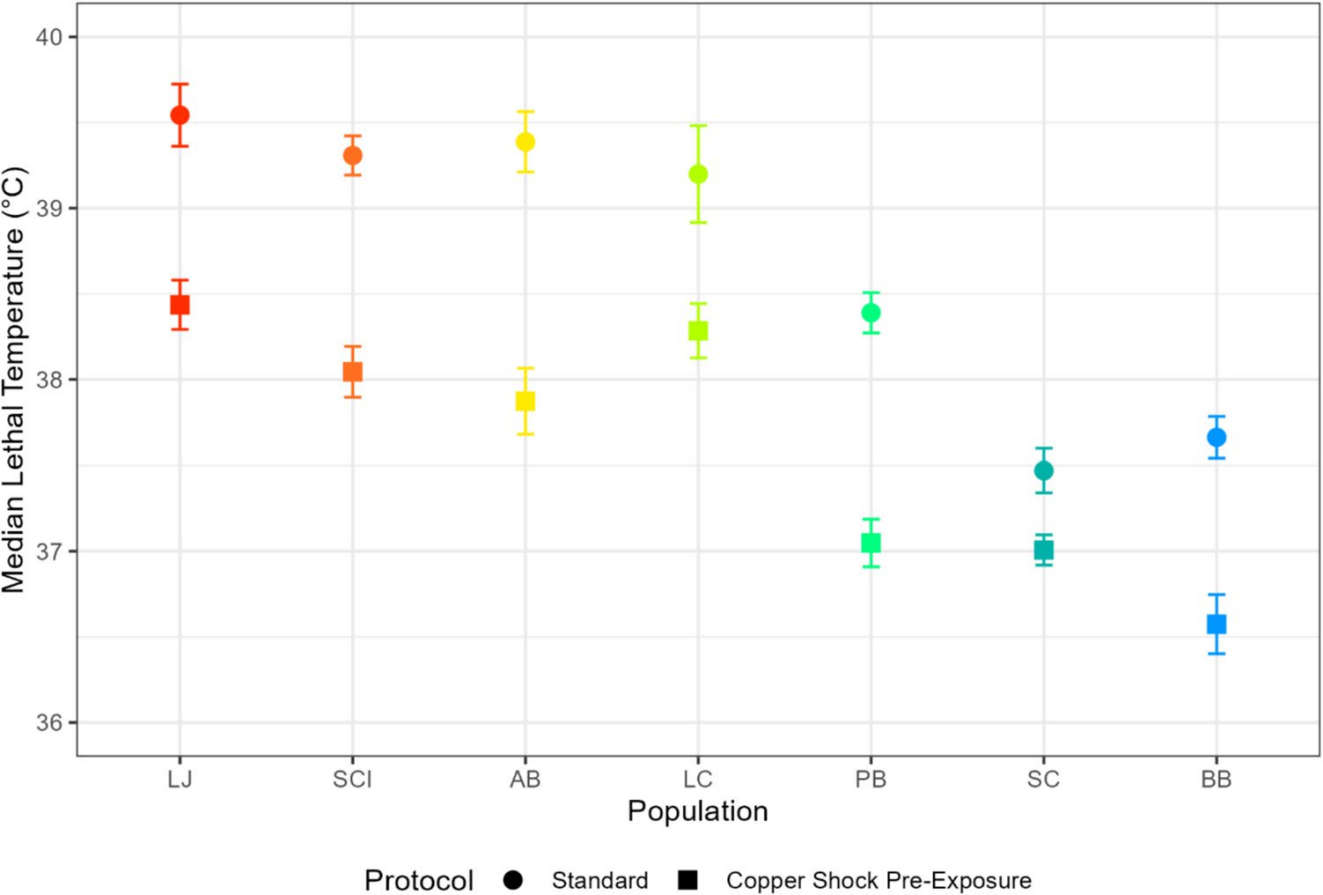
Median lethal temperature of seven *T. californicus* populations from standard thermal tests (circles) and from thermal tests that were preceeded by copper exposure (squares). Error bars represent the standard error of each LT50 value. BB, Bodega Bay; SC, Santa Cruz; PB, Pismo Beach; LC, Leo Carrillo; AB, Abalone Cove; SCI, Santa Catalina Island; LJ, La Jolla

**Fig. 5:**
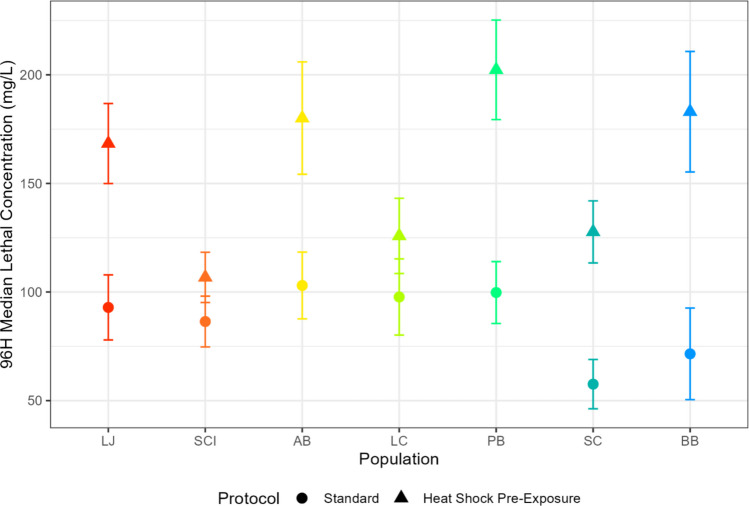
96-h median lethal concentrations (LC50s) of seven *T. californicus* populations from standard copper toxicity tests (circles) and from toxicity tests that were preceeded by heat exposure (triangles). Error bars represent the standard error of each LC50 value. BB, Bodega Bay; SC, Santa Cruz; PB, Pismo Beach; LC, Leo Carrillo; AB, Abalone Cove; SCI, Santa Catalina Island; LJ, La Jolla

In the sequential stressor experiments, thermal tolerance in all populations significantly decreased when exposed to copper prior to thermal testing (Fig. 4; Table S4). The reverse exposure experiments produced the opposite result, as copper tolerance significantly increased in all populations when the copepods experienced a heat shock prior to copper toxicity testing (Fig. 5; Table S4). To assess whether the magnitude of pre-exposure effects varied systematically across latitude, we also examined ΔLT50 and ΔLC50 (standard – pre-exposed) as a function of latitude and found that neither relationship was significant (Fig. S1).

### Differential gene expression

**Fig. 6:**
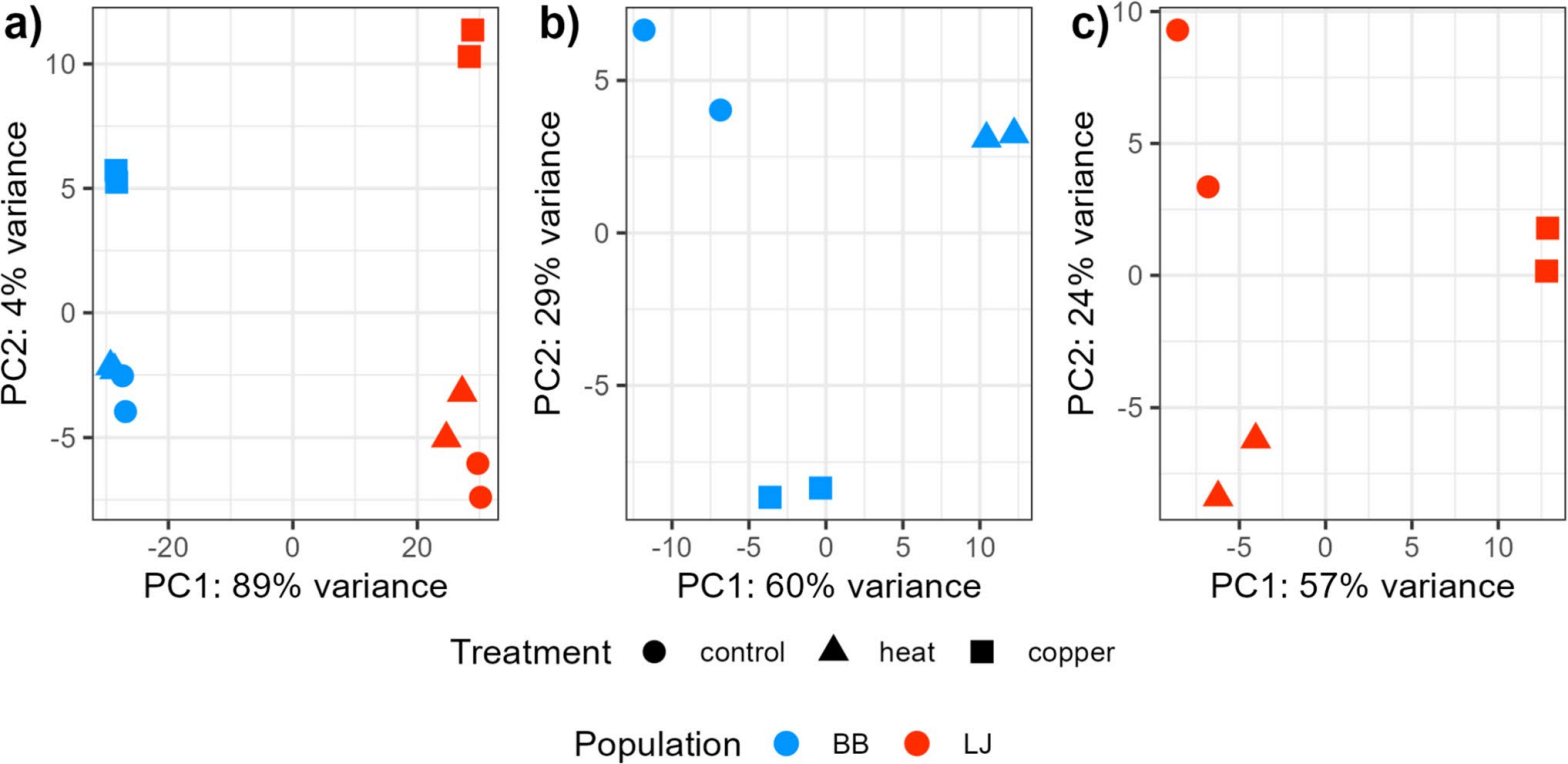
Principal component analysis (PCA) of the top 500 most variable genes from all 12 samples (**a**), and for the samples from each population considered separately (**b**: Bodega Bay, **c**: La Jolla). Point shape refers to a sample’s treatment, and point color refers to population. BB, Bodega Bay; LJ, La Jolla. Control = 24 h in 20 °C with no copper, Heat = 24 h in 35 °C with no copper, Copper = 24 h in 20 °C with 60 mg/L Cu

**Fig. 7:**
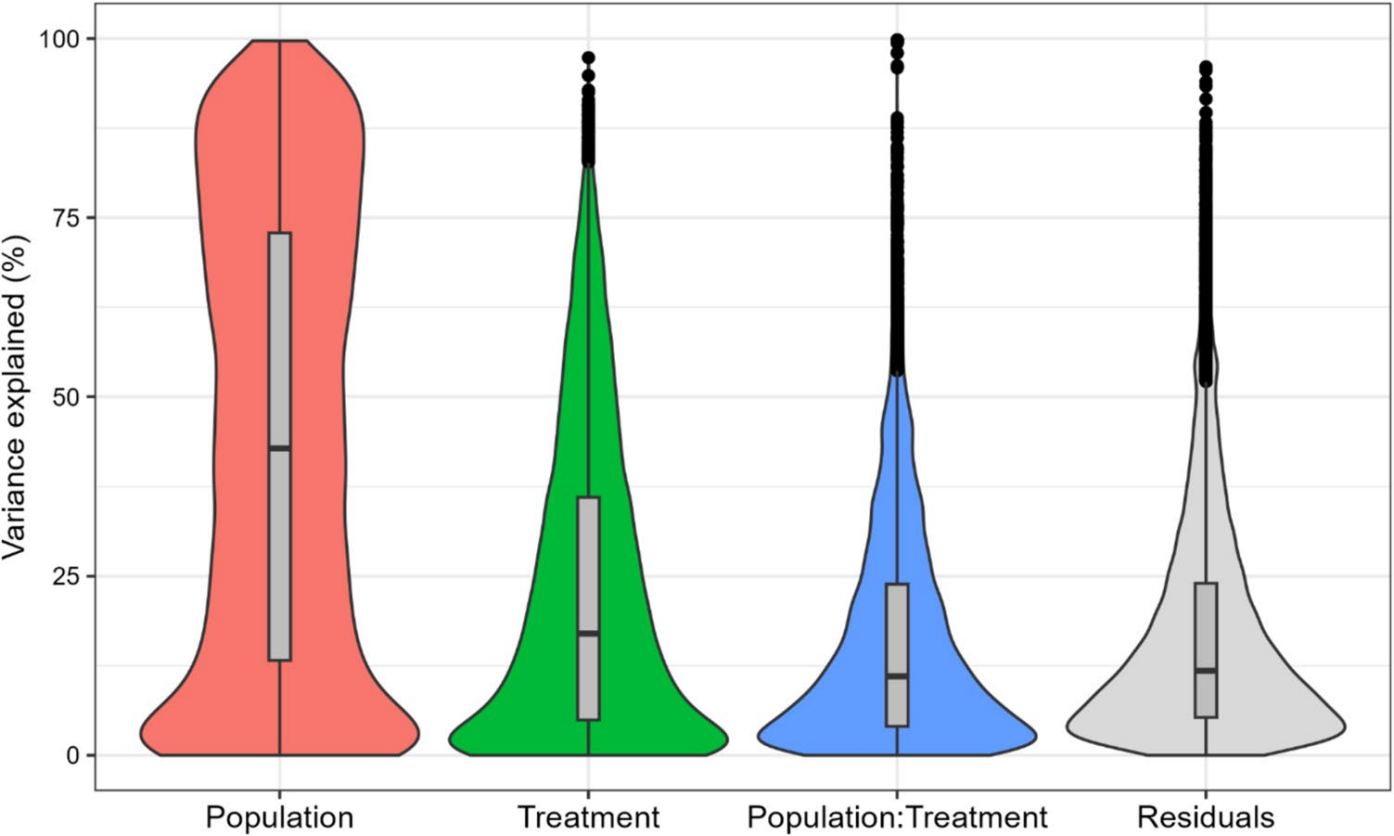
Violin plot showing fractions of variance in gene expression explained by population, treatment, population × treatment interaction, and model residuals

RNA-seq was conducted for 12 samples (2 replicates * (2 treatments + 1 control) * 2 populations) of pooled male copepods which yielded approximately 28.7 million 150 bp reads per sample. On average, 51.91% of Bodega Bay reads and 71.17% of La Jolla reads aligned to the San Diego reference genome (Table S5). Principal component analysis indicated that population was largely associated with PC1, which explained 89% of the gene expression variation between all 12 samples, while treatment primarily sorted on PC2, which explained just 4% of the variance (Fig. 6a). La Jolla’s gene expression in the heat treatment appeared to be more like the pattern seen in the control than in the copper treatment (Fig. 6c), while there was more distinct separation between the three treatment groups from Bodega Bay (Fig. 6b). Variance partitioning analysis indicated that on average, population accounted for 44% of the variation in gene expression, while treatment explained 23%, population × treatment interactions contributed 16%, and model residuals made up the remaining 17% (Fig. 7).

**Fig. 8:**
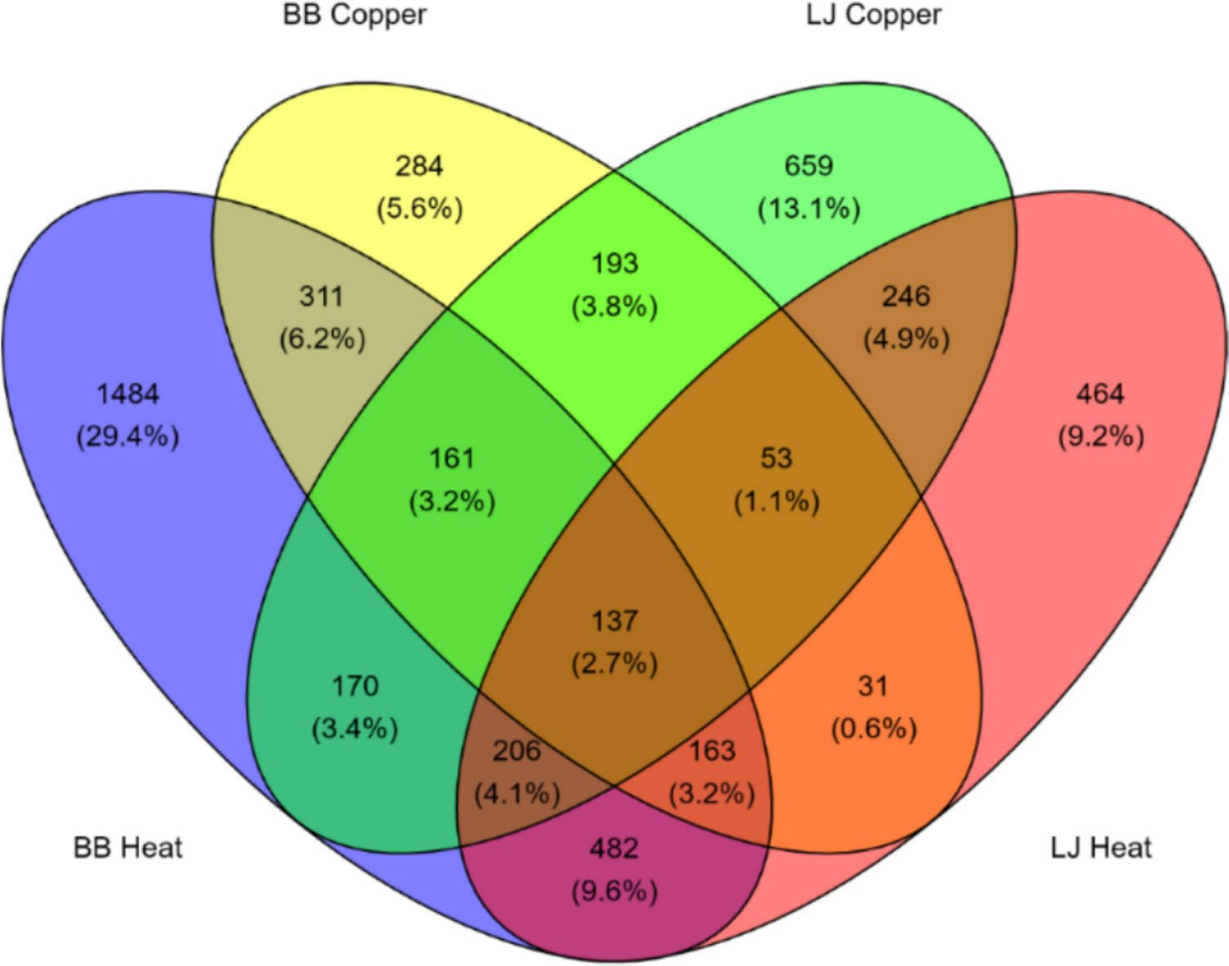
Venn diagram of the number of differentially expressed genes (Benjamini–Hochberg p < 0.1) shared between all population-treatment combinations. BB, Bodega Bay; LJ, La Jolla. Heat = 24 h in 35 °C with no copper, Copper = 24 h in 20 °C with 60 mg/L Cu

Relative to the controls, Bodega Bay differentially expressed 3114 genes in the heat treatment and 1333 during copper exposure, with 772 DEGs modified by both stressors in this population (Fig. 8). La Jolla differentially expressed more genes (1825) during copper exposure than heat (1782), and there were 642 DEGs shared in the two treatments (Fig. 8). Fisher’s exact tests indicated that these population differences in DEG counts were statistically significant for both heat and copper exposures (*p* < 2.2 × 10⁻^1^⁶). Across populations, 137 DEGs were shared in both treatments (Fig. 8; Table S7).

The antioxidant, heat shock protein, stress-induced chitin, and cuticle-associated genes that were differentially expressed in response to each treatment by both populations are listed in Table 1. Antioxidant genes were primarily upregulated in both treatments, while heat shock proteins and stress-induced genes were predominantly downregulated. Genes related to the cuticle and chitin metabolism were roughly equally up-and downregulated. The Bodega Bay heat treatment had the most DEGs (53) in these categories of all four groups, while the Bodega Bay copper treatment had only 29 DEGs. In contrast, La Jolla had more responsive DEGs in these categories in the copper treatment (37) than in the heat treatment (26). Of the 137 genes that were differentially expressed by both populations in both treatments, none was related to chitin or the cuticle, one was an antioxidant, five were heat shock proteins, and one was a stress-induced gene, and all genes in these categories were downregulated (Table S7).

**Table 1:** The number of differentially expressed antioxidant, heat shock protein, stress-induced chitin, and cuticle genes in each treatment

Category	Gene name	Bodega Bay				La Jolla			
		Heat		Copper		Heat		Copper	
		Up	Down	Up	Down	Up	Down	Up	Down
Antioxidant	Ascorbate peroxidase	0	1	1	0	1	0	0	0
	Catalase	1	0	0	0	1	0	0	0
	Glutathione peroxidase	1	0	0	0	1	0	0	0
	Glutathione s-transferase	11	0	0	0	4	0	4	2
	Mitogen-activated protein kinases	2	0	0	0	0	0	0	0
	Oxidoreductase	3	0	1	0	1	1	0	1
	Peroxiredoxin	1	0	1	0	3	0	4	0
	Superoxide dismutase	2	0	0	0	0	0	1	0
	Thioredoxin	0	3	0	3	1	1	1	3
	Thioredoxin domain-containing protein	0	1	0	1	0	1	1	1
Heat shock protein (HSP)	HSP beta-1	1	0	0	0	0	0	0	0
	HSP10	0	1	0	0	0	0	0	0
	HSP16.48/16.49	0	1	0	1	0	0	0	1
	HSP60	0	1	0	1	0	1	0	1
	HSP70	0	4	0	5	0	2	0	5
	HSP83	0	1	0	1	0	0	0	1
	HSP90	0	1	0	1	0	1	0	0
Stress-induced	Stress-70 protein	0	1	0	1	0	0	0	1
	Stress-induced-phosphoprotein 1-like	0	1	0	1	0	1	0	1
	Oxidative stress-induced growth inhibitor 1	1	0	0	0	0	0	0	1
Chitin	Chitinase	2	4	1	5	1	0	1	5
	Chitin deacetylase	4	2	2	2	1	0	0	2
	Chitin synthase	0	0	1	0	1	0	0	0
Cuticle	Cuticle protein 21	1	0	0	0	1	0	0	0
	Cuticle protein 27	0	0	0	0	1	0	0	0
	Cuticle protein 38	1	0	0	0	1	0	0	0
	Total responding genes	31	22	7	22	18	8	12	25

Up = Gene was upregulated relative to control

Down = Gene was downregulated relative to control

#### Gene ontology enrichment

**Fig. 9:**
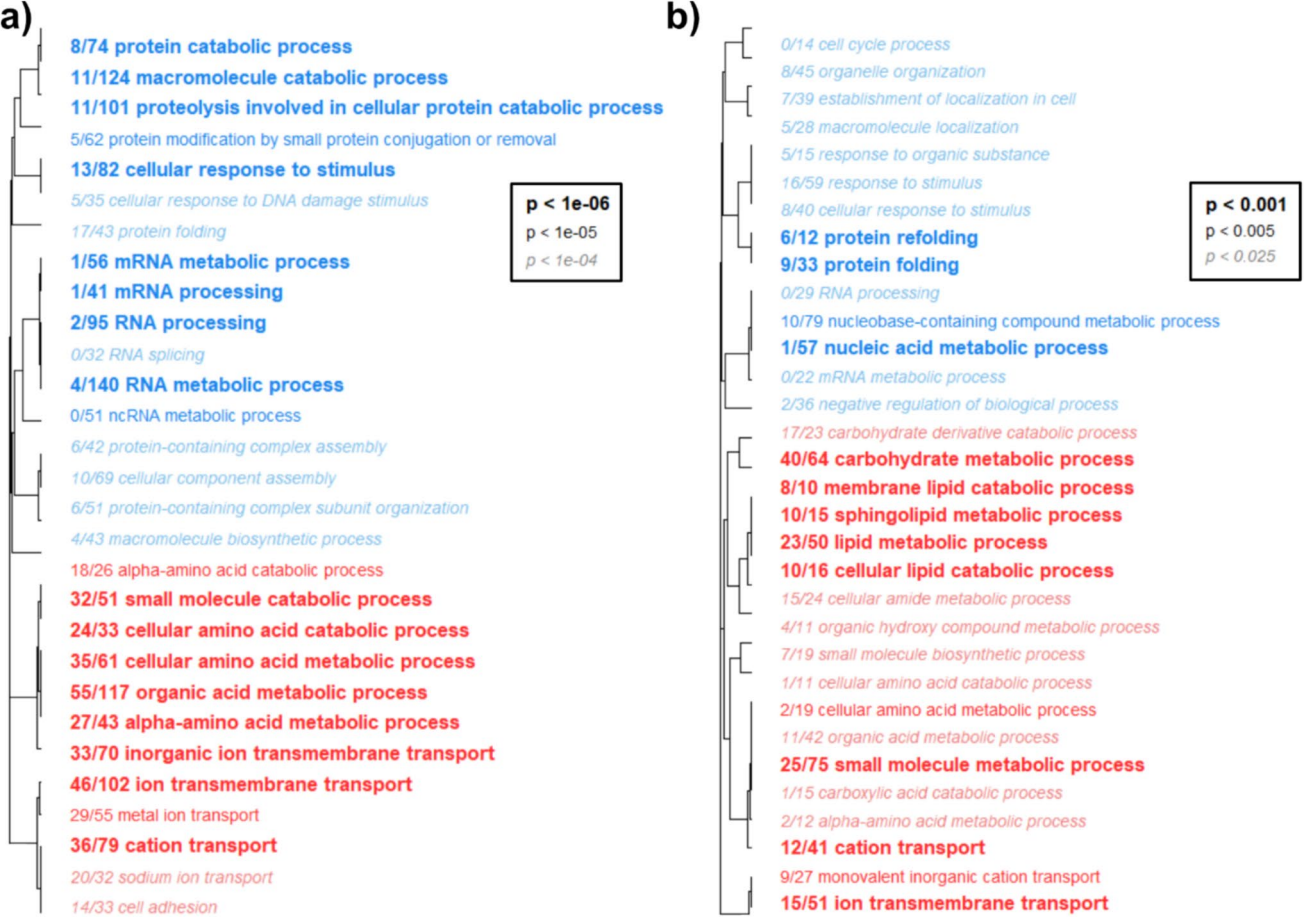
Hierarchical clustering of biological process (BP) gene ontology (GO) categories in Bodega Bay copepods exposed to heat (**a**) and copper (**b**). Font style indicates level of statistical significance, and colors indicate enrichment of categories with either upregulated (red) or downregulated (blue) genes. The fraction preceding each category name indicates the number of “good candidate” genes exceeding an absolute log2-fold change value of 1 relative to the total number of genes in the category

**Fig. 10:**
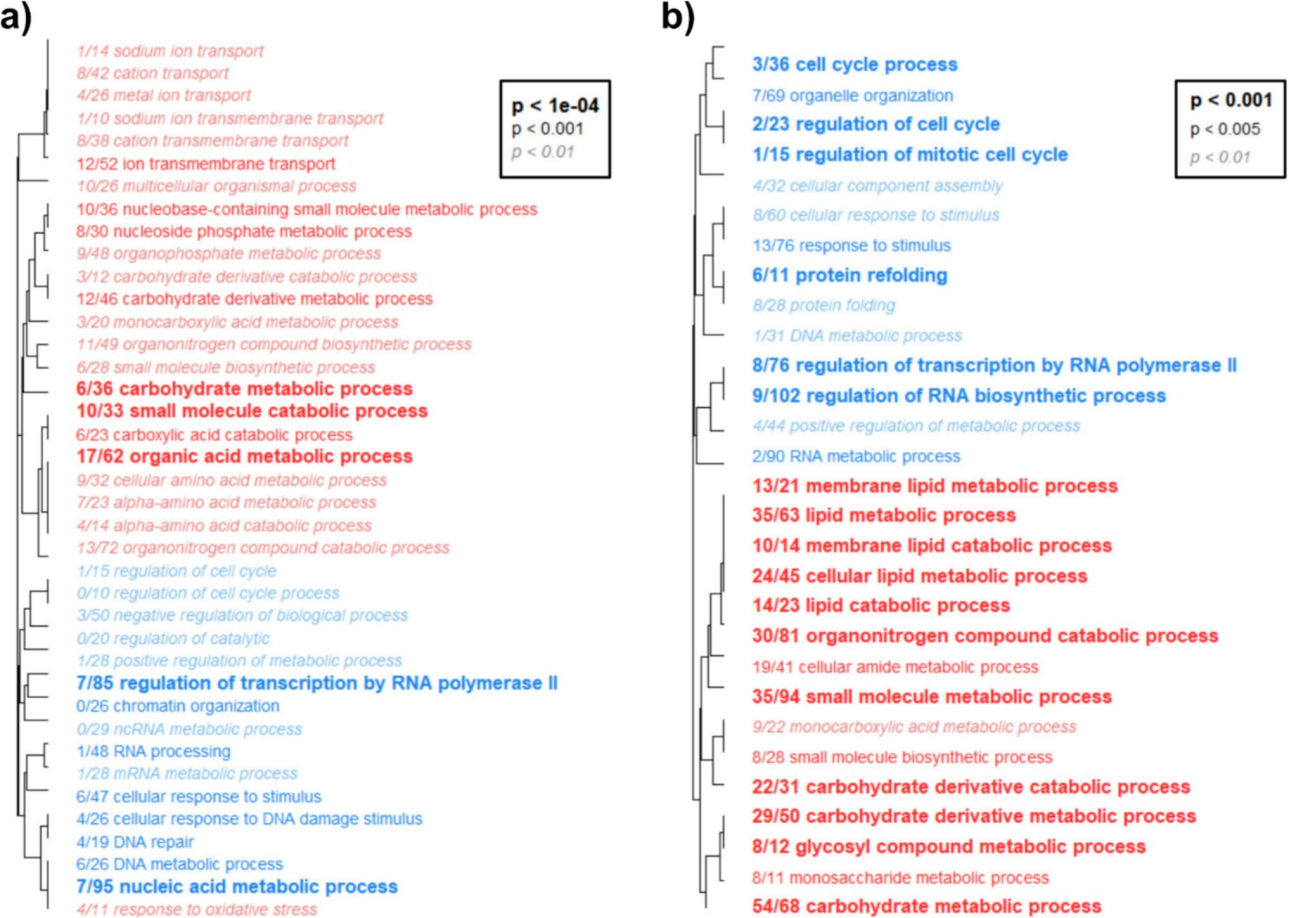
Hierarchical clustering of biological process (BP) gene ontology (GO) categories in La Jolla copepods exposed to heat (**a**) and copper (**b**). Font style indicates level of statistical significance, and colors indicate enrichment of categories with either upregulated (red) or downregulated (blue) genes. The fraction preceeding each category name indicates the number of “good candidate” genes exceeding an absolute log2-fold change value of 1 relative to the total number of genes in the category

During heat exposure, Bodega Bay animals largely upregulated biological processes related to amino acid metabolism and catabolism, as well as the transport of metals and ions (Fig. 9a). Copper exposure also induced upregulation of categories related to the metabolism of amino acids, carbohydrates, and lipids, as well as ion transport (Fig. 9b). GO categories downregulated by Bodega Bay during both treatments included processing of mRNA, protein folding, and organization, as well as cellular responses to stimuli (Fig. 9). La Jolla upregulated the metabolism and catabolism of amino acids and macromolecules in both treatments, while downregulating categories centered on the processing of nucleic acids and regulation of the cell cycle (Fig. 10).

## Discussion

### Latitudinal trends in acute thermal and copper tolerance

The results of our single stressor experiments align with the previously established latitudinal cline in thermal tolerance among *T. californicus* populations (Kelly et al. 2011; Leong et al. 2018; Willett 2010) and provide additional evidence for a similar cline in copper tolerance. In both cases, tolerance increases at lower latitudes, although this trend was only statistically significant for heat stress. However, the significant positive association between LT50 and LC50 values (Fig. 3c) suggests that latitude may partially explain the variation in copper tolerance among populations. While the relationship between temperature, latitude, and thermal tolerance is well-established in ectotherms (Sunday et al. 2010), the factors driving the corresponding latitudinal pattern in copper tolerance in *T. californicus* remain unclear. Although quadratic models marginally improved fit for the LT50-latitude and LT50-LC50 relationships (Table S3), the limited number of populations sampled here (*n* = 7) and their incomplete coverage of this species’ full latitudinal range constrain robust inference regarding nonlinear relationships. Notably, Leong et al. (2018) detected a nonlinear thermal cline in *T. californicus,* incorporating substantially broader geographic sampling of populations than considered here, suggesting that more extensive spatial coverage is required to resolve such patterns. Accordingly, we interpret our results as evidence of latitudinal trends in tolerance rather than definitive support for linear or nonlinear relationships.

Local adaptation to chemical pollution has been identified in a variety of species (Brown 1978; Klerks & Levinton 1989; Nacci et al. 2010), arising when populations in contaminated environments experience strong, sustained selective pressure for increased tolerance. However, in *T. californicus,* it remains unclear whether populations are exposed to sufficiently high levels of copper pollution to induce selection. For example, Sun et al. (2015) found similar copper concentrations in *Tigriopus* pools from Central and Southern California and that their corresponding populations could tolerate copper concentrations in the lab that greatly exceeded environmental levels. Because neither our study nor previous published work provides comprehensive measurements of copper in *T. californicus* pools, the extent to which natural copper gradients contribute to the observed tolerance patterns remains unresolved. Thus, in the absence of additional data on the spatial distribution of copper pollution, our results support the hypothesis that the variation in copper tolerance in *T. californicus* is driven by factors other than direct exposure. Given that many cellular stress response mechanisms are highly conserved (Kültz 2005), elevated copper tolerance in this species may in part reflect a correlated response to selection on thermal tolerance. Future studies that quantify copper concentrations in *T. californicus* splash pools and adjacent surface waters across the species’ broad range are essential to validate or refute this hypothesis.

### Unidirectional cross-protection between heat and copper

Our sequential exposure experiments revealed both cross-protection and cross-susceptibility interactions, depending on the order of exposure and stressor intensity. When copepods experienced a brief heat shock prior to copper testing, copper tolerance significantly increased across all populations, indicating a cross-protection interaction. Studies in other organisms have shown that a heat shock can increase tolerance of various stressors aside from copper (Bilyk et al. 2012; Neumann et al. 1994; Todgham et al. 2005), suggesting that an initial heat exposure can broadly prime conserved cellular defenses for subsequent challenges. Cross-protection is hypothesized to be driven in part by transcriptional frontloading (Collins et al. 2020), in which constitutive gene expression shifts to maintain physiological tolerance mechanisms that respond to common environmental stressors (Barshis et al. 2013). Frontloading is associated with reduced transcriptional plasticity during stress exposure, owed to the higher baseline expression of genes that respond to the stressor (Barshis et al. 2013). Reduced transcriptional plasticity has been identified as a byproduct of heat adaptation in *T. californicus* (Kelly et al. 2017), which may further contribute to the cross-protection outcome we observed.

In contrast, exposing copepods to copper before thermal testing reduced heat tolerance in all populations (Fig. 4). This response is consistent with cross-susceptibility (Todgham & Stillman 2013), the phenomenon in which exposure to one stressor increases vulnerability to a subsequent stressor. Cross-susceptibility occurs when the physiological and energetic demands imposed by the initial stressor limit an organism’s ability to mount a response to the secondary stressor, typically by either damaging physiological systems or depleting energy reserves (Rodgers & Gomez Isaza 2022). The likelihood of cross-susceptibility depends in part on the magnitude and duration of the initial stressor, as an elongated or high intensity exposure is likely to undermine future stress tolerance. We hypothesize that the severity of the initial copper exposure in our copper shock experiments contributed to the cross-susceptibility result. By the same reasoning, substantially higher or longer heat exposures than the treatment used here (1 h at 35 °C) could also overwhelm cellular defenses and prevent heat-induced cross-protection in a heat first-sequence.

Although no mortalities were observed during initial copper exposure (1 h at 60 mg/L Cu), data from the single stressor experiments suggests that this treatment was a more severe stress event than the heat shock (1 h at 35 °C). Specifically, in the standard heat stress experiments, none of the populations experienced mortality at 35 °C, whereas after 24 h in 60 mg/L Cu, test organism populations decreased on average by 27% (Table S6). It is possible that a cross-protection effect could manifest with a lower copper shock concentration or a longer recovery period before to heat testing. Alternatively, the cross-susceptibility induced by this sequence of stressors may mean that the overlap between the copper and heat stress response mechanisms is less robust than initially hypothesized.

### Population predominantly determines gene expression

Our RNA-seq analysis highlights the dominant role of population in determining gene expression in *T. californicus,* with treatment effects inducing comparatively subtle shifts. This pattern is consistent across both PCA and variance partitioning analyses (Figs. 6–7). Previous work with *T. californicus* has identified substantial genetic divergence between natural populations (Burton 1998; Rawson & Burton 2006; Willett & Ladner 2009), with greater differentiation occurring between southern populations (south of Oregon to Baja California) than northern (Oregon to Alaska) (Edmands 2001). Within this framework, Bodega Bay and La Jolla are both considered southern populations, meaning that significant genetic differences between them are to be expected.

Notably, Bodega Bay exhibited a stronger transcriptional response to heat stress than La Jolla, differentially expressing 3114 genes compared to 1782. This finding is consistent with previous comparative transcriptomics work in *Tigriopus* (Kelly et al. 2017; Li et al. 2020) and other systems (Barshis et al. 2013; Chen et al. 2019; Gleason & Burton 2015), which found that less tolerant types tend to show greater induction of stress DEGs, suggesting that the more tolerant types either possess a more efficient response mechanism or experience a lower level of stress. In contrast, the copper treatment did not follow this pattern. Instead, La Jolla, the more copper-tolerant population (Fig. 3b), differentially expressed more genes (1825) than Bodega Bay (1333). This discrepancy may have resulted from differences in exposure length, as the copepods utilized for RNA-seq experienced a shorter copper exposure than those in the acute toxicity tests, potentially affecting the magnitude of transcriptional responses.

### Similar modulation of oxidative defenses and biological processes by both stressors

Although both populations exhibited distinct transcriptional profiles under copper and heat (Fig. 6; Fig. S2), broad similarities in their responses to the two stressors suggest potential mechanisms could contribute to cross-protection. For instance, both stressors altered the expression of antioxidant genes (Table 1), which play a crucial role in mitigating oxidative stress. Genes from the glutathione s-transferase (GST) enzyme family, which catalyze the conjugation of the reduced form of glutathione with xenobiotic and hydrophilic molecules (Hayes & Strange 1995), were particularly active (Table 1), pointing to major involvement of the glutathione system in the responses to both stressors. High glutathione activity confers protection from oxidative damage (Kidd 1997), so the priming of glutathione metabolism by heat shock could provide a major boost to defenses during subsequent copper exposure.

Additionally, both stressors caused copepods to downregulate cell cycle processes, nucleic acid metabolism and transcription, likely as a protective measure to prevent further damage to DNA (Fuse et al. 1996; Niskanen et al. 2015; Shackelford et al. 2000). The catabolism of amino acids and large macromolecules was upregulated simultaneously (Figs. 9, 10), potentially increasing available energetic resources for secondary stress responses while reducing the energetic cost of cell cycle regulation. The upregulation of ion transport during heat stress (Figs. 9a, 10a) may also contribute to cross-protection by priming cells to more efficiently translocate excess copper ions during toxicity. In addition, genes associated with cuticle formation responded to both stressors; however, their potential role in cross-protection is unclear because the specific function of the cuticle in the heat stress response remains uncertain (Harada & Burton 2020; Schoville et al. 2012).

Unexpectedly, we also found that heat shock proteins (Table 1) and protein folding processes (Figs. 9, 10) were largely downregulated by both stressors, contrasting with previous studies in *Tigriopus* that identified HSP upregulation during chemical or oxidative stress (Kim et al. 2014; Li et al. 2023, 2020). Because we extracted RNA after 24 h of exposure, a later time point than other studies that sampled with 6 h of stress initiation (Graham & Barreto 2019; Harada & Burton 2019; N. Li et al. 2020; Schoville et al. 2012), it is possible that HSP production had already peaked at the time of sampling. Our results may therefore reflect reestablished HSP homeostasis rather than the initial induction phase. If HSPs do undergo a rapid surge during stress exposure, this process could be a major contributor to cross-protection by reducing the need for additional protein synthesis during secondary stress exposure and allowing resources to be diverted elsewhere. Future studies that measure HSP abundance and activity across multiple time points during exposure will be essential to determine how transcriptional patterns correspond to functional responses.

## Conclusions

In the present study, we provide further insights into the interplay between thermal and copper stress responses in the intertidal copepod *T. californicus* and highlight the importance of stressor sequence and intensity in shaping cross-protection outcomes. Our acute stress experiments identified correlated latitudinal trends in acute thermal and copper tolerance, suggesting that shared physiological mechanisms may underlie adaptation to both stressors, although the specific drivers of copper tolerance remain unresolved. Cross-protection was evident when copepods were exposed first to heat and then copper, while the reverse scenario of copper exposure prior to heat stress negatively impacted tolerance. Gene expression responses to stress were highly population-specific, although there was evidence to suggest that both copper and heat similarly modulated the antioxidant defense system and triggered metabolic reprogramming that could generate cross-protection. Given that climate change is increasing both the frequency of extreme heat events and the mobilization of pollutants in coastal environments, our findings suggest that *T. californicus* populations may exhibit differential resilience depending on their prior stress exposure and genetic background. Future work examining additional populations, environmental copper levels, and the transcriptional dynamics of stress responses over time will be critical to fully understanding the mechanisms driving cross-tolerance and susceptibility in this system. Broadly, these results underscore that idea that predictions of biological responses to climate change and pollution must account for the sequence and intensity of stressor exposure. Because many organismal stress pathways are conserved, similar interaction effects are likely to occur across taxa, which can have critical consequences for ecological forecasting and environmental management.

## Supplementary Information

Below is the link to the electronic supplementary material.

ESM 1 (DOCX 380 KB)

## Data Availability

Acute tolerance data and RNA-seq sample read counts are available on Zenodo at 10.5281/zenodo.18602925. Raw sequencing data from our twelve RNA-seq samples are available on the National Center for Biotechnology Information’s (NCBI) Sequence Read Archive (SRA) under BioProject Accession Number PRJNA1420951.
