# Supplementary material for: Heat exposure improves acute copper tolerance in the intertidal copepod Tigriopus californicus
Source: Environ Sci Pollut Res Int. 2026 Mar 25;33(12):5640–56. doi: 10.1007/s11356-026-37621-2 (PMC13091868; doi:10.1007/s11356-026-37621-2)
Supplement: Supplementary file 1 — (DOCX 380 KB) [file 11356_2026_37621_MOESM1_ESM.docx]

**Heat exposure improves acute copper tolerance in the intertidal copepod *Tigriopus californicus***

Alice L. Coleman^1*^ and Suzanne Edmands^1^

^1^University of Southern California Department of Biological Sciences, 3616 Trousdale Parkway #130, Los Angeles, CA, 90089, USA

*Corresponding author (Email: [a.coleman1914@gmail.com](mailto:a.coleman1914@gmail.com))

Submitted to *Environmental Science and Pollution Research*

## **Supplementary Materials**


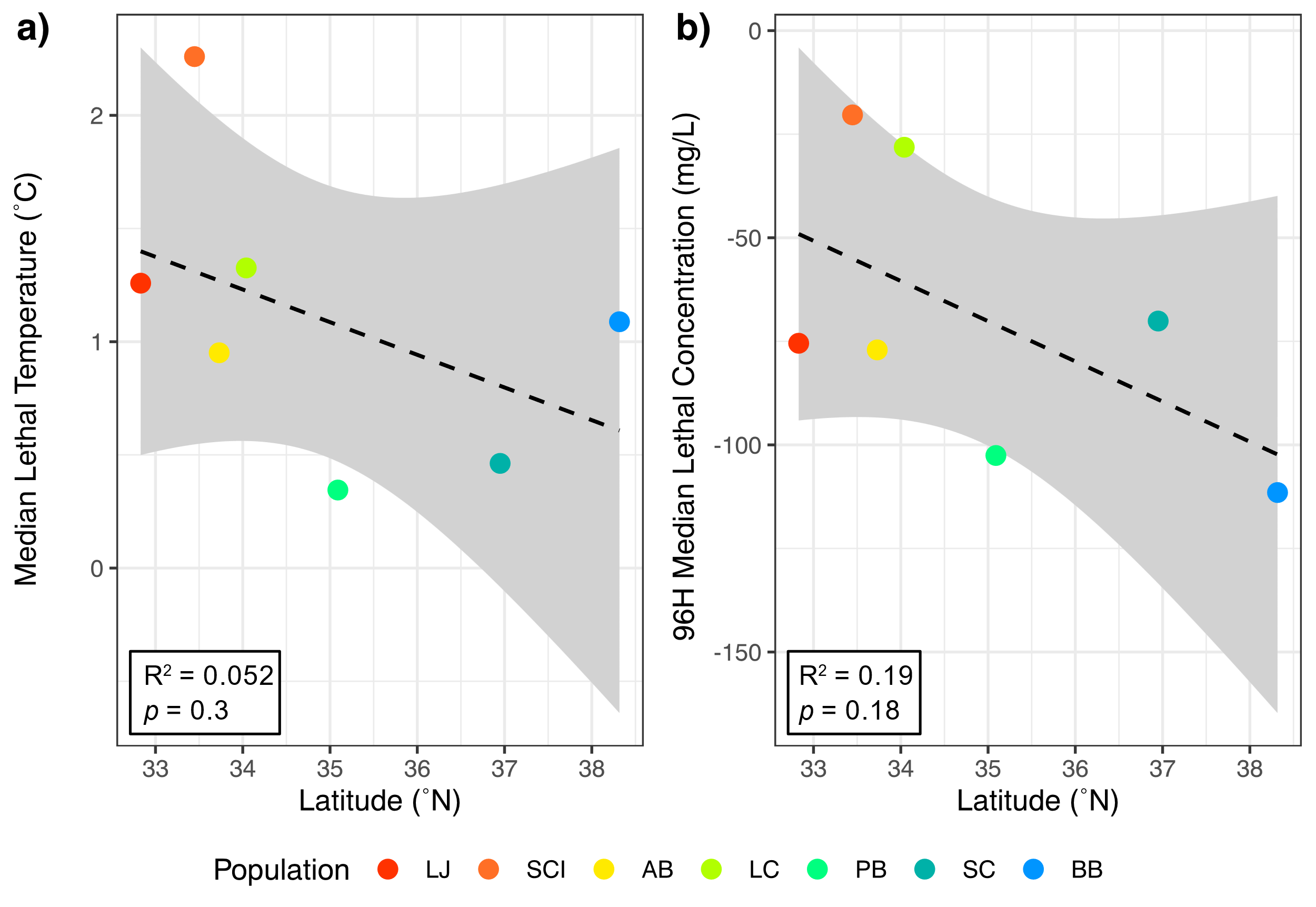


**Figure S1:** ΔLT50 and ΔLC50 across the latitudinal gradient. Relationship between ΔLT50 (standard LT50 – copper shock LT50) and latitude (a). Relationship between ΔLC₅₀ (standard LC50 – heat shock LC50) and latitude (b). The shading around each trend line represents its 95% confidence interval. Solid trend lines are statistically significant (*p* < 0.05), dashed trend lines are not (*p* > 0.05). BB = Bodega Bay, SC = Santa Cruz, PB = Pismo Beach, LC = Leo Carrillo, AB = Abalone Cove, SCI = Santa Catalina Island, LJ = La Jolla


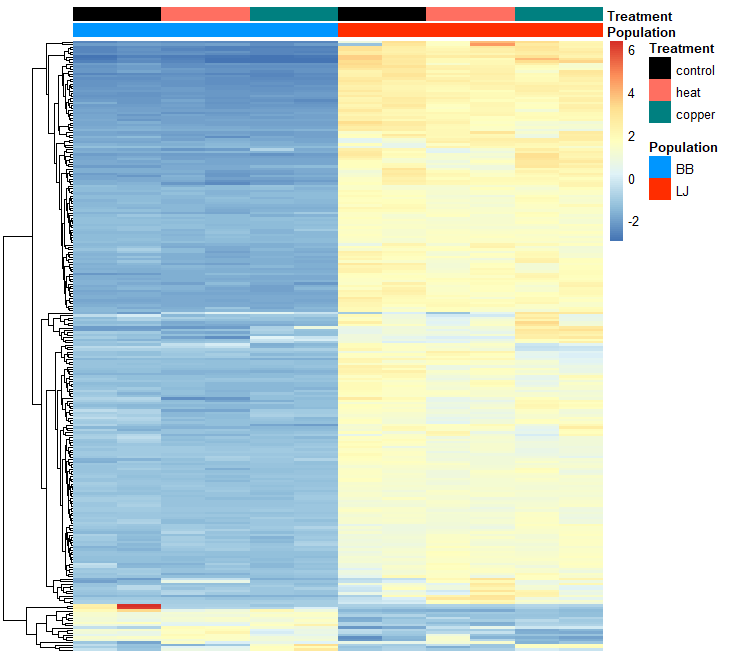


**Figure S2**: Heatmap of the rlog transformed expression values of the top 250 most variable genes across all groups. BB= Bodega Bay, LJ = La Jolla. Control = 24 hrs in 20°C with no copper, Heat = 24 hrs in 35°C with no copper, Copper = 24 hrs in 20°C with 60 mg/L Cu.

**Table S1:** Approximate sampling coordinates for each *Tigriopus californicus* population

| **Population** | **Latitude (°N)** | **Longitude (°W)** |
| --- | --- | --- |
| Bodega Bay (BB) | 38.32 | -123.1 |
| Santa Cruz (SC) | 36.95 | -122.1 |
| Pismo Beach (PB) | 35.09 | -120.7 |
| Leo Carrillo (LC) | 34.04 | -118.9 |
| Abalone Cove (AB) | 33.73 | -118.4 |
| Santa Catalina Island (SCI) | 33.45 | -118.5 |
| La Jolla (LJ) | 32.83 | -117.3 |

**Table S2**: RNA concentration, quantity and RNA Integrity Number (RIN) of the twelve samples used for RNA-sequencing as reported by Admera Health (New Jersey, USA)

| **Sample ID** | **Population** | **Treatment** | **Concentration  (ng/uL)** | **Total Quantity  (ng)** | **RNA Integrity Number  (RIN)** |
| --- | --- | --- | --- | --- | --- |
| BA1 | Bodega Bay (BB) | Control | 15.8 | 459.1 | 8.4 |
| BA2 | Bodega Bay (BB) | Control | 15.8 | 411.3 | 8.3 |
| BA3 | Bodega Bay (BB) | Heat | 17 | 425 | 8.6 |
| BA4 | Bodega Bay (BB) | Heat | 10.5 | 252 | 8.3 |
| BA5 | Bodega Bay (BB) | Copper | 16.3 | 406.3 | 8.3 |
| BA6 | Bodega Bay (BB) | Copper | 20.7 | 537.2 | 8.3 |
| LA1 | La Jolla (LJ) | Control | 15.8 | 473.4 | 7.9 |
| LA2 | La Jolla (LJ) | Control | 20.9 | 605.5 | 8.3 |
| LA3 | La Jolla (LJ) | Heat | 21.7 | 630.2 | 9 |
| LA4 | La Jolla (LJ) | Heat | 17.5 | 508.7 | 8.9 |
| LA5 | La Jolla (LJ) | Copper | 32.1 | 929.5 | 8.6 |
| LA6 | La Jolla (LJ) | Copper | 22.8 | 662.1 | 9.7 |

**Table S3**: Comparison of linear and quadratic models fitted to tolerance and latitude datasets

| **Dataset** | **Model Type** | | | | | |
| --- | --- | --- | --- | --- | --- | --- |
|  | **Linear** | | | **Quadratic** | | |
|  | **Adjusted R^2^** | ***p*-value** | **AIC** | **Adjusted R^2^** | ***p*-value** | **AIC** |
| LT50 vs Latitude (Fig. 3A) | 0.89 | 8.4E-04 | 5.9 | 0.91 | 3.3E-03 | 4.7 |
| LC50 vs Latitude (Fig. 3B) | 0.46 | 0.056 | 59 | 0.36 | 0.18 | 60 |
| LT50 vs LC50 (Fig. 3C) | 0.57 | 0.031 | 57 | 0.83 | 0.013 | 51 |

**Table S4**: Pairwise comparisons of LT50 (°C) and LC50 (mg/L) values between standard and sequential exposure treatments for each population based on ratio tests

| **Population** | **Endpoint** | **Standard Estimate (°C or mg/L)** | **Pre-exposed estimate (°C or mg/L)** | **Test statistic** | ***p*-value** |
| --- | --- | --- | --- | --- | --- |
| Bodega Bay (BB) | LT50 | 37.7 | 36.6 | 190 | <2.2E-16 |
| Bodega Bay (BB) | LC50 | 71.5 | 183 | 320 | <2.2E-16 |
| Santa Cruz (SC) | LT50 | 37.5 | 37 | 110 | <2.2E-16 |
| Santa Cruz (SC) | LC50 | 57.6 | 128 | 193 | <2.2E-16 |
| Pismo Beach (PB) | LT50 | 38.4 | 37 | 277 | <2.2E-16 |
| Pismo Beach (PB) | LC50 | 99.8 | 202 | 41.6 | <2.2E-16 |
| Leo Carillo State Beach (LC) | LT50 | 39.2 | 38.2 | 110 | <2.2E-16 |
| Leo Carillo State Beach (LC) | LC50 | 97.7 | 126 | 119 | <2.2E-16 |
| Abalone Cove (AB) | LT50 | 39.4 | 37.9 | 224 | <2.2E-16 |
| Abalone Cove (AB) | LC50 | 103 | 180 | 656 | <2.2E-16 |
| Santa Catalina Island (SCI) | LT50 | 39.3 | 37 | 259 | <2.2E-16 |
| Santa Catalina Island (SCI) | LC50 | 86.4 | 107 | 61.5 | <2.2E-16 |
| La Jolla (LJ) | LT50 | 39.5 | 38.4 | 187 | <2.2E-16 |
| La Jolla (LJ) | LC50 | 92.9 | 168 | 579 | <2.2E-16 |

**Table S5**: Number of raw reads and percentage of 150 bp reads mapped to the San Diego reference genome in each sample

| **Sample Name** | **Population** | **Treatment** | **Total Reads** | **Alignment Rate** |
| --- | --- | --- | --- | --- |
| BA1 | Bodega Bay | Control | 21,115,893 | 52.93% |
| BA2 | Bodega Bay | Control | 27,883,234 | 50.36% |
| BA3 | Bodega Bay | Heat | 30,539,955 | 53.43% |
| BA4 | Bodega Bay | Heat | 30,291,769 | 53.66% |
| BA5 | Bodega Bay | Copper | 33,581,001 | 49.88% |
| BA6 | Bodega Bay | Copper | 29,273,615 | 51.21% |
| LA1 | La Jolla | Control | 25,311,298 | 62.68% |
| LA2 | La Jolla | Control | 32,840,962 | 71.33% |
| LA3 | La Jolla | Heat | 33,848,509 | 73.17% |
| LA4 | La Jolla | Heat | 29,258,125 | 72.07% |
| LA5 | La Jolla | Copper | 23,944,229 | 69.05% |
| LA6 | La Jolla | Copper | 26,983,968 | 78.72% |

**Table S6**: Copepod mortalities after a thermal stress test at 35°C and after 24 hours in 60 mg/L Cu

| **Population** | **Treatment** | **Sample Size** | **Mortalities** |
| --- | --- | --- | --- |
| Bodega Bay | 35°C | 12 | 0 |
| Santa Cruz | 35°C | 24 | 0 |
| Pismo Beach | 35°C | 36 | 0 |
| Leo Carrillo | 35°C | 36 | 0 |
| Abalone Cove | 35°C | 12 | 0 |
| Santa Catalina Island | 35°C | 48 | 0 |
| La Jolla | 35°C | 12 | 0 |
| Bodega Bay | 60 mg/L Cu | 72 | 12 |
| Santa Cruz | 60 mg/L Cu | 114 | 28 |
| Pismo Beach | 60 mg/L Cu | 47 | 13 |
| Leo Carrillo | 60 mg/L Cu | 48 | 18 |
| Abalone Cove | 60 mg/L Cu | 96 | 35 |
| Santa Catalina Island | 60 mg/L Cu | 90 | 29 |
| La Jolla | 60 mg/L Cu | 113 | 24 |

**Table S7**: Genes that were differentially expressed in all four treatment-population combinations

| Gene Symbol | Gene Name | Bodega Bay | | La Jolla | |
| --- | --- | --- | --- | --- | --- |
|  |  | Heat | Copper | Heat | Copper |
| LOC131876887 | hepatic lectin-like | up | up | up | up |
| LOC131876909 | trimethylamine monooxygenase-like | up | up | up | down |
| LOC131877019 | uncharacterized LOC131877019 | up | up | up | up |
| LOC131877154 | 26S proteasome non-ATPase regulatory subunit 8-like | down | down | down | down |
| LOC131877195 | heat shock 70 kDa protein cognate 4-like | down | down | down | down |
| LOC131877444 | uncharacterized LOC131877444 | up | up | up | up |
| LOC131877456 | phytanoyl-CoA dioxygenase, peroxisomal-like | up | up | up | up |
| LOC131877494 | proton channel OtopLc-like | up | up | up | up |
| LOC131877497 | lysosomal proton-coupled steroid conjugate and bile acid symporter SLC46A3-like | up | up | up | up |
| LOC131877542 | uncharacterized LOC131877542 | up | up | up | up |
| LOC131877579 | UDP-glycosyltransferase UGT5-like | up | up | up | up |
| LOC131877758 | E3 ubiquitin-protein ligase HUWE1-like | down | down | down | down |
| LOC131877879 | von Willebrand factor A domain-containing protein 2-like | up | up | up | up |
| LOC131877888 | uncharacterized LOC131877888 | up | up | up | up |
| LOC131877935 | GTP cyclohydrolase 1-like | up | up | up | up |
| LOC131878120 | cyanophycinase-like | up | up | up | up |
| LOC131878146 | zinc finger protein 345-like | down | down | down | down |
| LOC131878271 | forkhead box protein D1-like | up | up | up | up |
| LOC131878619 | uncharacterized LOC131878619 | up | up | up | up |
| LOC131878931 | mannan endo-1,4-beta-mannosidase-like | down | up | up | up |
| LOC131879070 | uncharacterized LOC131879070 | up | up | up | up |
| LOC131879170 | alkaline phosphatase 4-like | down | up | down | down |
| LOC131879250 | uncharacterized LOC131879250 | up | up | up | up |
| LOC131879386 | uncharacterized LOC131879386 | up | up | up | up |
| LOC131879440 | m7GpppN-mRNA hydrolase-like | down | down | down | down |
| LOC131879525 | glutamate decarboxylase-like | up | up | up | up |
| LOC131879562 | sodium/potassium-transporting ATPase subunit alpha-B-like | up | up | up | up |
| LOC131879648 | perlucin-like | up | up | up | up |
| LOC131879822 | protein disulfide-isomerase-like | up | up | up | up |
| LOC131879932 | poly(3-hydroxybutyrate) depolymerase-like | down | up | up | up |
| LOC131880156 | N-acetylated-alpha-linked acidic dipeptidase 2-like | up | up | up | up |
| LOC131880287 | aldo-keto reductase family 1 member B1-like | up | down | up | down |
| LOC131880393 | uncharacterized LOC131880393 | down | down | down | down |
| LOC131880395 | uncharacterized LOC131880395 | down | down | up | up |
| LOC131880542 | NF-X1-type zinc finger protein NFXL1-like | down | down | down | down |
| LOC131881090 | zwei Ig domain protein zig-4-like | up | up | up | up |
| LOC131881189 | tubulin beta chain-like | up | up | up | up |
| LOC131881262 | brachyurin-like | down | up | up | up |
| LOC131881263 | brachyurin-like | down | up | up | up |
| LOC131881304 | uncharacterized LOC131881304 | up | up | down | down |
| LOC131881410 | ecdysone receptor-like | up | down | up | down |
| LOC131881557 | pre-mRNA-splicing factor ATP-dependent RNA helicase PRP16-like | down | down | down | down |
| LOC131881669 | cartilage oligomeric matrix protein-like | up | up | up | up |
| LOC131881683 | innexin inx2-like | up | up | up | up |
| LOC131881773 | sucrase-isomaltase, intestinal-like | down | up | up | up |
| LOC131882171 | elongation of very long chain fatty acids protein 7-like | up | up | up | up |
| LOC131882396 | sphingosine kinase 1-like | up | up | up | up |
| LOC131882573 | cullin-2-like | down | down | down | down |
| LOC131882656 | nuclear receptor subfamily 1 group I member 3-like | up | down | up | down |
| LOC131882734 | uncharacterized LOC131882734 | up | up | up | up |
| LOC131882756 | uncharacterized LOC131882756 | up | up | up | up |
| LOC131882851 | heat shock protein 60A-like | down | down | down | down |
| LOC131883101 | electrogenic sodium bicarbonate cotransporter 1-like | up | up | up | up |
| LOC131883102 | uncharacterized LOC131883102 | up | up | up | up |
| LOC131883190 | heat shock 70 kDa protein 1-like | down | down | down | down |
| LOC131883226 | anti-sigma-I factor RsgI6-like | down | up | up | up |
| LOC131883257 | phosphatidylethanolamine-binding protein homolog F40A3.3-like | up | up | up | up |
| LOC131883348 | uncharacterized LOC131883348 | up | up | up | up |
| LOC131883421 | soma ferritin-like | down | down | down | down |
| LOC131883585 | maltase 1-like | down | up | down | up |
| LOC131884124 | long-chain-fatty-acid--CoA ligase ACSBG2-like | up | up | up | up |
| LOC131884219 | erythroid differentiation-related factor 1-like | down | down | down | down |
| LOC131884450 | hemocyte protein-glutamine gamma-glutamyltransferase-like | up | up | up | up |
| LOC131884767 | sodium-dependent glucose transporter 1-like | up | up | up | up |
| LOC131884796 | exosome complex exonuclease RRP44-like | down | down | down | down |
| LOC131884862 | pancreatic triacylglycerol lipase-like | down | up | up | up |
| LOC131884964 | uncharacterized LOC131884964 | up | up | up | up |
| LOC131884990 | facilitated trehalose transporter Tret1-like | up | up | up | down |
| LOC131885169 | purine nucleoside phosphorylase-like | up | up | up | up |
| LOC131885266 | protein c-Fos-like | up | down | up | down |
| LOC131885392 | aquaporin-like | up | up | up | up |
| LOC131885413 | uncharacterized LOC131885413 | up | up | up | up |
| LOC131885448 | proteasome subunit beta type-2-like | down | down | down | down |
| LOC131885609 | protein ultraspiracle homolog | up | up | up | up |
| LOC131885767 | peptidyl-prolyl cis-trans isomerase E-like | down | down | down | down |
| LOC131885782 | uncharacterized LOC131885782 | up | up | up | up |
| LOC131885938 | activator of 90 kDa heat shock protein ATPase homolog 1-like | down | down | down | down |
| LOC131885956 | uncharacterized LOC131885956 | down | down | down | down |
| LOC131886042 | rhomboid-related protein 1-like | down | down | up | down |
| LOC131886100 | uncharacterized LOC131886100 | down | down | up | down |
| LOC131886208 | phenoloxidase-activating factor 2-like | up | up | up | up |
| LOC131886565 | F-box/LRR-repeat protein 14-like | up | up | up | up |
| LOC131886705 | acetylcholine receptor subunit alpha-like | down | up | down | up |
| LOC131887236 | uncharacterized LOC131887236 | down | up | down | up |
| LOC131887246 | serine protease inhibitor 42Dd-like | up | up | up | up |
| LOC131887366 | endoglucanase E-4-like | down | up | up | up |
| LOC131887389 | uncharacterized LOC131887389 | down | down | down | down |
| LOC131887398 | switch-associated protein 70-like | up | up | up | up |
| LOC131887516 | phenoloxidase-activating factor 1-like | up | up | up | up |
| LOC131887784 | uncharacterized LOC131887784 | up | up | up | up |
| LOC131888374 | beta-1,3-galactosyltransferase 1-like | up | up | up | up |
| LOC131888540 | myosin-11-like | down | down | down | down |
| LOC131888543 | uncharacterized LOC131888543 | down | down | up | down |
| LOC131888698 | heat shock protein Hsp-16.48/Hsp-16.49-like | down | down | down | down |
| LOC131889100 | legumain-like | up | up | up | up |
| LOC131889587 | 26S proteasome regulatory subunit 10B | down | down | down | down |
| LOC131889658 | T-complex protein 1 subunit eta-like | down | down | down | down |
| LOC131889700 | sarcoplasmic calcium-binding proteins I, III, and IV-like | up | up | up | up |
| LOC131889779 | uncharacterized LOC131889779 | up | up | up | up |
| LOC131889812 | uncharacterized LOC131889812 | up | up | up | up |
| LOC131889939 | uncharacterized LOC131889939 | up | up | up | up |
| LOC131889974 | uncharacterized LOC131889974 | up | up | up | up |
| LOC131889976 | E3 ubiquitin-protein ligase UBR5-like | down | down | down | down |
| LOC131890012 | dehydrogenase/reductase SDR family member 7-like | up | up | up | up |
| LOC131890038 | uncharacterized LOC131890038 | up | up | up | up |
| LOC131890082 | glutaminase liver isoform, mitochondrial-like | up | up | up | up |
| LOC131890125 | cleavage stimulation factor subunit 3-like | down | down | down | down |
| LOC131890141 | sodium-dependent proline transporter-like | up | up | up | up |
| LOC131890204 | uncharacterized LOC131890204 | up | up | up | up |
| LOC131890381 | procathepsin L-like | up | up | up | up |
| LOC131890401 | carbonic anhydrase-like | up | up | up | up |
| LOC131890772 | protein Skeletor | up | up | up | up |
| LOC131891097 | stress-induced-phosphoprotein 1-like | down | down | down | down |
| LOC131891304 | neprilysin-2-like | up | up | up | up |
| LOC131891407 | uncharacterized LOC131891407 | up | up | up | up |
| LOC131891578 | uncharacterized LOC131891578 | up | down | up | down |
| LOC131891581 | leukocyte elastase inhibitor-like | up | up | up | up |
| LOC131891602 | aldo-keto reductase family 1 member B1-like | up | up | up | up |
| LOC131891872 | eukaryotic translation initiation factor 4E-binding protein 1-like | up | up | up | up |
| LOC131892192 | uncharacterized oxidoreductase MexAM1_META1p0182-like | down | down | down | down |
| LOC131892305 | uncharacterized LOC131892305 | up | up | up | up |
| LOC131892439 | uncharacterized LOC131892439 | down | up | up | up |
| LOC131892594 | uncharacterized LOC131892594 | up | up | up | up |
| LOC131892771 | DNA topoisomerase 2-alpha-like | down | down | down | down |
| LOC131892816 | protein 5NUC-like | up | up | up | up |
| LOC131892901 | proteasome subunit alpha type-4-like | down | down | down | down |
| LOC131892917 | endoplasmic reticulum chaperone BiP-like | down | down | down | down |
| LOC131893007 | uncharacterized LOC131893007 | down | down | down | down |
| LOC131893080 | uncharacterized LOC131893080 | down | down | down | down |
| LOC131893202 | DNA replication licensing factor mcm4-A-like | down | down | down | down |
| LOC131893218 | uncharacterized LOC131893218 | up | up | up | up |
| LOC131893225 | uncharacterized LOC131893225 | up | up | up | up |
| LOC131893370 | uncharacterized LOC131893370 | down | down | down | down |
| LOC131893372 | uncharacterized LOC131893372 | down | down | down | down |
| LOC131893447 | mitochondrial glycine transporter A-like | up | up | up | up |
| LOC131893449 | uncharacterized LOC131893449 | down | down | down | down |
| LOC131893645 | probable cytochrome P450 6a14 | up | up | up | up |

Up = Gene was upregulated relative to control

Down = Gene was downregulated relative to control
